# Ontogeny of Expression and Activity of Digestive Enzymes and Establishment of *gh*/*igf1* Axis in the Omnivorous Fish *Chelon labrosus*

**DOI:** 10.3390/ani10050874

**Published:** 2020-05-18

**Authors:** Neda Gilannejad, Verónica de las Heras, Juan Antonio Martos-Sitcha, Francisco J. Moyano, Manuel Yúfera, Gonzalo Martínez-Rodríguez

**Affiliations:** 1 Instituto de Ciencias Marinas de Andalucía (ICMAN-CSIC), 11519 Puerto Real, Cádiz, Spain; veronica.delasher@alum.uca.es (V.d.l.H.); manuel.yufera@icman.csic.es (M.Y.); gonzalo.martinez@csic.es (G.M.-R.); 2 Department of Biology, Faculty of Marine and Environmental Sciences, Instituto Universitario de Investigación Marina (INMAR), Campus de Excelencia Internacional del Mar (CEI-MAR), University of Cádiz, 11519 Puerto Real, Cádiz, Spain; juanantonio.sitcha@uca.es; 3 Departamento de Biología y Geología, Facultad de Ciencias, Campus de Excelencia Internacional del Mar (CEI-MAR), Universidad de Almería, La Cañada de San Urbano, 04120 Almería, Spain; fjmoyano@ual.es

**Keywords:** *Chelon labrosus*, digestive function, enzyme activity, gene expression, ontogeny, somatotropic factors

## Abstract

**Simple Summary:**

Thick-lipped grey mullet (*Chelon labrosus*) feeds on the lowest trophic levels during adult stages, for which it is considered a viable candidate for an economically and environmentally sustainable aquaculture. Similar to most of marine fish species, *C. labrosus* produce a large number of eggs, leading to morphologically and anatomically larvae that are not completely mature and have to pass through substantial differentiation and development in their functional systems to acquire adult features. Therefore, the study of the development of digestive tract and of the growth regulation can provide useful information to adapt the feeding protocols and rearing conditions to the physiological requirements at each stage. This work aimed to evaluate the early ontogeny of key digestive enzymes and somatotropic factors at biochemical and/or transcriptional levels. Our results evidenced that maturation of the digestive system and acquisition of the adult mode of digestion occurs around 60 to 70 days post hatch (dph), when starch or other low-cost carbohydrate-based compounds could be used in formulated diets at increasing levels. Furthermore, our results implied an independent expression of the studied somatotropic genes during the first 40 dph and establishment of a functional growth hormone/insulin-like growth factor 1 axis from 50 dph onward.

**Abstract:**

Thick-lipped grey mullet (*Chelon labrosus*) is a candidate for sustainable aquaculture due to its omnivorous/detritivorous feeding habit. This work aimed to evaluate its digestive and growth potentials from larval to early juvenile stages. To attain these objectives the activity of key digestive enzymes was measured from three until 90 days post hatch (dph). Expression of genes involved in digestion of proteins (*try2*, *ctr*, *pga2*, and *atp4a*), carbohydrates (*amy2a*), and lipids (*cel* and *pla2g1b*), together with two somatotropic factors (*gh* and *igf1*) were also quantified. No chymotrypsin or pepsin activities were detected. While specific activity of trypsin and lipase were high during the first 30 dph and declined afterward, amylase activity was low until 57 dph and increased significantly beyond that point. Expression of *try2*, *ctr*, *amy2a*, and *cel* increased continuously along development, and showed a peak at the end of metamorphosis. Expression of *pla2g1b*, *pga2* and *atp4a* increased until the middle of metamorphosis and decreased afterwars. Most of these trends contrast the usual patterns in carnivorous species and highlight the transition from larvae, with high protein requirements, to post-larvae/juvenile stages, with omnivorous/detritivorous feeding preferences. Somatotropic genes, *gh* and *igf1*, showed approximately inverse expression patterns, suggesting the establishment of the Gh/Igf1 axis from 50 dph.

## 1. Introduction

Thick-lipped grey mullet (*Chelon labrosus*) has several characteristics that make it an interesting candidate for diversification and sustainability of aquaculture production [1,2,3]. First, it has omnivorous/detritivorous feeding habits and therefore feeds on the lowest trophic levels [2]. Thick-lipped grey mullet larvae and post-larvae mainly feed on zooplankton and small crustaceans, while the natural diet in adults is based on benthic diatoms, epiphytic algae, small invertebrates, and detritus [4]. Besides, as other mullets, *C. labrosus* has a high osmoregulatory capacity, allowing it to inhabit a broad range of salinities without negative effects on growth rate [1,2]. Moreover, in contrast to most other mullet species, it can be reared in captivity during the whole life cycle through induced or natural spawning.

Similar to most marine fish species, thick-lipped grey mullet produces a large number of eggs leading to morphologically and anatomically immature larvae [2]. Therefore, functional systems of the newly hatched larvae pass through substantial differentiation and development to acquire adult features. Within this context, the study of the development of the digestive tract and of the growth regulation are considered key indicators providing information that can be useful to adapt feeding protocols and rearing conditions to the physiological requirements of each stage.

The ontogeny of digestive system has been traditionally addressed using anatomical, histological, and biochemical approaches. These methods have been complemented more recently with molecular biology techniques [5,6]. Although the study of the ontogeny of digestive enzymes at transcriptional level can provide useful information due to its high accuracy and specificity even for different gene isoforms [7], it has been carried out in relatively few species [6]. Additionally, even fewer studies have assessed the ontogeny of digestive system using simultaneously molecular and biochemical techniques [5,8,9]. Such studies can provide a better insight into transcriptional/translational relations of the digestive machinery and can help to understand to what extent the enzymatic hydrolysis of macronutrients is programmed, activated, and/or modulated [6].

Early development of digestive, visual, and other organs of the thick-lipped grey mullet has been studied using histochemistry approaches [10]. According to this study, most functional structures and organs of the digestive system (i.e., pancreatic zymogen granules, liver, etc.) were differentiated early after hatching and were developed before mouth opening. Moreover, the relative activity of cytosolic versus brush border enzymes measured in larvae of *C. labrosus* reared in mesocosm suggested a precocious intestinal maturation [1,3]. These authors therefore, considered the possibility of an early application of co-feeding and weaning in this species at eight or from 14 to 20 days post hatch (dph).

Despite continuous fish growth during lifetime, growth rates could vary along different stages. Growth pattern of *C. labrosus* during early development has been characterized by a primary arrested somatic growth during first days after hatching, followed by a further acceleration [1,2]. Understanding the factors involved in the regulation of growth and development is important for aquaculture production since it provides useful insights on the possibilities to influence the different pathways. Similar to mammals, the endocrine regulation of growth in fish is mainly produced by the growth hormone (Gh)/insulin-like growth factor (Igf) axis [11]. Both Gh and Igf1 are known to play a critical role during early fish development [12,13]. The *gh* gene is mainly expressed in pituitary, but also in extra-pituitary tissues [14]. In contrast to adult fish, in which it is principally expressed in the liver, Igf1 has been detected in diverse tissues during larval stages, and therefore, besides Gh-mediated growth regulation, it plays a wide range of biological roles including muscle development, osmoregulation, and hematopoiesis [15,16].

The aim of present work was to evaluate the early ontogeny of key digestive enzymes and somatotropic factors at biochemical and/or transcriptional levels during the initial development of the thick-lipped grey mullet. Unlike most of the available literature, in which only short periods of 30–45 dph are usually addressed [6], this work encompasses a wider period, from hatching until three months of life. To evaluate the digestive functionality, the activity of several key digestive enzymes involved in the digestion of proteins, lipids and carbohydrates (trypsin, chymotrypsin, pepsin, lipase, and amylase) were measured. Additionally, the expression of genes controlling these proteases (*trypsinogen 2*, *try2*; *chymotrypsinogen*, *ctr*; *pepsinogen a2*, *pga2*; and proton pump or gastric H^+^/K^+^-ATPase, *atp4a*), lipases (bile salt activated lipase or *carboxyl ester lipase*, *cel*; and *pancreatic phospholipase a2*, *pla2g1b*), and carbohydrases (*pancreatic alpha amylase*, *amy2a*) was quantified. To address the growth potential, two key somatotropic factors (*growth hormone*, *gh*; and *insulin like growth factor 1*, *igf1*) were also determined.

## 2. Materials and Methods

### 2.1. Fish Rearing and Sampling Protocol

This study was carried out in compliance with the Guidelines of the European Union Council (2010/63/EU) and Spanish legislation for the use of laboratory animals, with approval of the Bioethics Committee of the Spanish National Research Council for project “Optimization of the maturation and spawning processes in the mullet *Chelon labrosus*.”

*C. labrosus* fertilized eggs were obtained from natural spawning in captivity of nine females and 11 males, in I.E.S. Els Alfacs (Tarragona, Spain), and transferred to the ICMAN experimental facilities (REGA ES110280000311) one day after spawning. Newly hatched larvae, three days after spawning, were distributed and reared in three cylindro-conical tanks of 250 L. The day of hatching was considered as 0 dph. During the rearing period, temperature and pH ranged from 18.6 °C to 18.7 °C and 7.6 to 7.9, respectively. Salinity and photoperiod were constant at 35 ppt and LD 12:12, respectively.

**Figure 1:**
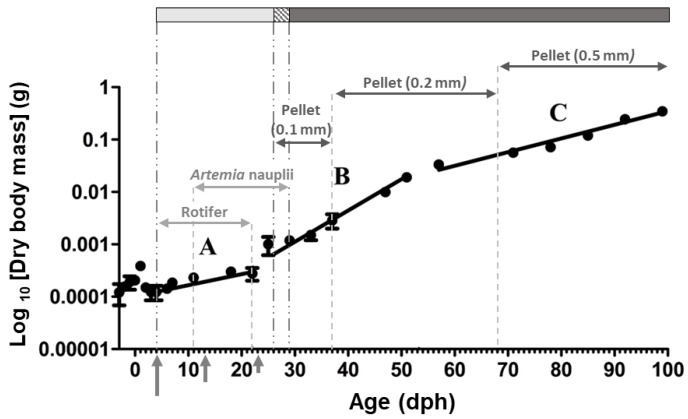
Growth pattern (Log_10_-transformed dry body mass) and feeding protocol for *Chelon labrosus* larvae utilized in the present study. Periods with different growth rates are indicated by letters A, B, and C (modified from Sarasquete et al. [10]). Long (4 dph), medium (13 dph), and short (23 dph) vertical arrows indicate mouth opening, onset of exclusively exogenous feeding, and proliferation of gastric glands, respectively. Light grey, dashed, and dark grey bars on top of the graph, represent periods with live prey, co-feeding, and inert diet, respectively.

Mouth opening and exclusively exogenous feeding (total absorption of yolk sac) occurred at four days post-hatching (dph) and 13 dph, respectively. From 0 dph to 29 dph, tanks were supplied with phytoplankton (*Nannochloropsis gaditana* and *Isochrysis galbana*). After mouth opening until 22 dph, larvae were fed rotifers (*Brachionus plicatilis*, 5 preys mL^−1^). From 11 dph, fish were supplied with *Artemia* nauplii (*Artemia salina*, 0.3–0.5 prey mL^−1^). From 20 dph to 29 dph, nauplii were gradually replaced with micro algae enriched meta-nauplii (1 prey mL^−1^). During the experimental period, live prey was supplied ad libitum. The commercial diet (Gemma, Skretting, Burgos, Spain) was initially supplied four times a day (starting from 09:00 a.m.) at 26 dph, being the only food offered to the fish from 29 dph onward (Figure 1). Proximate composition of the diet is detailed in Supplementary Table S1.

A total of 14 sampling points were chosen at different stages during larval and juvenile development according to the key ontogeny events defined by histochemical data presented by Sarasquete et al. [10]; before mouth opening (3 dph), at mouth opening (4 dph), during slow (8–21 dph), fast (28–50 dph), and medium growth (57–92 dph) periods (Figure 1; Supplementary Table S2). Sampling was carried out randomly, just at the beginning of the light period and before the first feeding (at 09:00). From each sampling point, 6 to 15 individuals (2 to 5 replicates per tank) were processed for each one of the enzyme activity and gene expression analyses. Fish were anaesthetized in MS-222 (Sigma-Aldrich; Buchs, Switzerland) and then rinsed with distilled water. For enzymes activity measurements, *C. labrosus* individuals were freeze-dried, to determine their dry body mass, and were then stored at −20 °C until being processed. For gene expression analysis, anesthetized fresh samples were preserved in an appropriate volume (1/10 *w*/*v*) of RNAlater^®^ (Invitrogen Life Technologies; Waltham, MA, USA), and after 24 h at 4 °C, were stored at −20 °C until total RNA isolation was performed.

### 2.2. Cloning and Gene Expression Analyses

This manuscript follows the ZFIN Zebrafish Nomenclature Guidelines for gene and protein names and symbols (https://wiki.zfin.org/display/general/ZFIN+Zebrafish+Nomenclature+Conventions).

#### 2.2.1. Molecular Cloning

To develop the molecular tools required for gene expression analyses, *beta actin* and *bile salt-activated lipase* full-length cDNAs, as well as *pancreatic phospholipase A2* and *gastric proton pump* partial cDNAs were amplified and cloned in *C. labrosus*. In brief, total RNA was extracted from the whole larvae using NucleoSpin^®^ XS kit (Macherey-Nagel) for individuals weighting less than 3 mg (until 9 dph), and NucleoSpin^®^ RNA II kit (Macherey-Nagel) for larger individuals. RNA quantity was measured using a Qubit^®^ 2.0 Fluorometer (Invitrogen, life Technologies) with Qubit^®^ RNA BR Assay Kit (Molecular Probes^®^, Life technologies), whereas RNA quality was assessed in a Bioanalyzer 2100 with the RNA 6000 Nano kit (Agilent Technologies, LifeSciences; Santa Clara, CA, USA). Only samples with an RNA Integrity Number (RIN) higher than 8.0, which is indicative of clean and intact RNA, were used for further analysis. For each sampling point, 6–10 individuals were processed. For reverse transcription, 250 ng of total RNA were used in a final volume of 20 µL using the qScript™ cDNA synthesis kit (Quanta BioSciences; Beverly, MA, USA). Besides, in order to increase the chance of cloning, cDNA was synthesized from a pool of RNA samples (from 3 dph to 78 dph) with the highest quality (RIN > 9.5).

For cloning the intermediate fragments of *cel* and *pla2g1b* in *C. labrosus*, degenerate primers were designed from conserved regions of full-length cDNA sequences of other teleost (Supplementary Tables S3 and S4; Supplementary Figures S1 and S2). In order to obtain the *atp4a* primary fragment, degenerate primers previously described for *Acipenser persicus* [17] were used. Besides, due to the high length of this gene, an elongation strategy toward the 3′ end of the cDNA sequence was adopted. For this, a specific forward primer, designed in the intermediate sequence, and three nested reverse degenerate primers for *A. persicus* [17], overlapping a minimum of 1259 base pairs (bp), were used (Supplementary Table S3; Supplementary Figure S3). In all these amplifications, the pooled cDNA sample served as template in the PCR reactions.

In order to clone the 5′- and 3′-ends of *actb* and *cel* in *C. labrosus*, gastrointestinal tract (GIT) cDNA library, constructed by Pujante et al. [18], was used as template in PCR reactions. For *actb*, quantitative real-time PCR (qPCR) forward and reverse primers from Pujante et al. [18], designed at the partial sequence available in the Genbank (AY836368.1), were used to obtain 3′-ends and 5′-ends, respectively. For *cel*, specific primers were designed from the intermediate primary sequence. In both cases, gene specific primers were run in combination with the pBluescript SK (–) Vector-specific universal primers, M13 Reverse and T3, as forward primers, to obtain the 5′-end and with M13 Forward (–20) and T7, as reverse primers, to obtain the 3′-end. The alignment between the primary partial sequences and the 5′-end and 3′-end sequences resulted in a minimum of 191 and 290 bp and 82 and 421 bp overlapping fragment for *actb* and *cel*, respectively (Supplementary Table S3; Supplementary Figures S1 and S4).

Primers used in this study were designed with the help of Primer3 software v. 0.4.0 (available at http://bioinfo.ut.ee/primer3-0.4.0/) and were synthetized by IDT^®^. PCR reactions were carried out in a Mastercycler^®^proS (Eppendorf; Hamburg, Germany) using Horse-Power™ Taq DNA Polymerase (Canvax Biotech; Córdoba, Spain) or Q5^®^ High-Fidelity DNA Polymerase (New England, BioLabs^®^ Inc.; Ipswich, MA, USA). PCR cycling protocols were 94 °C, 5 min; [95 °C, 30 s; 60 °C, 30 s; 72 °C, 1 min/kb] × 30 cycles; 72 °C, 10 min, or 98 °C, 30 s; [98 °C, 10 s; 60 °C, 20 s; 72 °C, 30 s/kb] × 35 cycles; 72 °C, 2 min, respectively. PCR products were run in agarose gels stained with GelRed™ (Biotium™; Fremont, CA, USA). Bands with expected size were purified using the NucleoSpin^®^ Gel and PCR Clean-up Kit (Macherey-Nagel; Düren, Germany), cloned using the CloneJET PCR Cloning Kit (Fermentas, Life Sciences; Waltham, MA, USA), and sequenced in BIOARRAY (Elche, Spain). Sequences for full-length cDNAs were assembled using the algorithm merger in EMBOSS explorer (available at http://www.bioinformatics.nl/emboss-explorer/).

#### 2.2.2. Gene Quantification Using Quantitative Real-Time PCR

Relative gene quantification was performed with the ΔΔC_T_ method [19], in a Mastercycler^®^epgradient S Realplex^2^ and the Realplex software v.2.2 (Eppendorf; Hamburg, Germany) with automated threshold and walking baseline cycle threshold (Ct) values. Results were normalized to β-actin, as the reference gene, due to its low variability among all the analyzed samples. A calibrator sample, cDNA pool of all the samples, was measured in all the plates to correct for inter-assay errors. qPCR reactions were performed in semi-skirted twin tec real-time PCR plates 96 (Eppendorf; Hamburg, Germany) covered with adhesive Masterclear real-time PCR Film (Eppendorf; Hamburg, Germany), with 5 ng of cDNA (assumed from RNA input), sense and antisense primers, and PerfeCTa™ SYBR^®^ Green FastMix™ (Quanta BioSciences; Beverly, MA, USA), in a final volume of 10 µL. The PCR protocol was as follows: 95 °C, 5 min; [95 °C, 15 s; 60 °C, 30 s] × 40 cycles; melting curve (60–95 °C, 20 min). Prior to analysis of the samples, for each gene, the cDNA pool was used to optimize the qPCR conditions using different primer combinations, a temperature gradient for annealing temperatures (55–60 °C), and primers concentrations (100 nM, 200 nM and 400 nM). Besides, a serial dilution of 1:10 (from 5 ng to 500 fg, using a 10 mM Tris•HCl-0.1 mM EDTANa_2_, pH 8.0 buffer) of the cDNA pool was used to check the efficiency of the amplification and R^2^ of the points in the calibration curve. In order to ensure the absence of primer-dimers and genomic DNA, control reactions with RNase free water and RNA were included in the curves. Quantification of *pga2*, *try2*, *ctr*, and *amy2a* was performed using the qPCR primers designed by Pujante et al. [20]. All the qPCR oligonucleotides, their position in their corresponding sequences, their final concentration in the reaction, amplification efficiency and R^2^ of the calibration curve, amplicon size, and accession number of the sequences are shown in Table 1.

### 2.3. Enzyme Activity Analyses

Enzyme extracts were prepared by manual homogenization of each individual larva in an appropriate volume of distilled water (200–750 µL, depending on fish size), followed by centrifugation at 19,000× *g* for 10 min at 4 °C (5810R; Eppendorf; Hamburg, Germany). The supernatants were separated in aliquots and kept at −20 °C until being used for enzyme activity measurements. For each sampling point, 7–13 individuals were processed.

Different fluorogenic substrates were used to evaluate activities of the digestive enzymes. For this, assays were carried out in microplates as detailed in Rotllant et al. [21]. Trypsin and chymotrypsin activities were evaluated using Boc-Gln-Ala-Arg-7-methylcoumarin hydrochloride (B4153; Sigma-Aldrich^®^; Buchs, Switzerland) and Ala-Ala-Phe-7-amido-4-methylcoumarin (Sigma-Aldrich^®^; Buchs, Switzerland, A3401), diluted in dimethyl sulfoxide (DMSO) to a final concentration of 20 μM, respectively. Assay mixture used 5 μL of substrate, 10 μL of the enzyme extract and 190 μL of buffer (200 mM borate + 100 mM CaCl_2_ pH 8 for trypsin and 200 mM Tris + 40 mM CaCl_2_ pH 8 for chymotrypsin), being fluorescence measured at 380/440 nm (excitation/emission). Pepsin activity was measured using EnzChek^®^ Protease Assay Kit (E33651; Molecular Probes^TM^; Waltham, MA, USA). This kit uses as substrate a fluorescent labeled casein dissolved in phosphate-buffered saline (pH 7.2) and then diluted in 200 mM glycine + HCl buffer (pH 3.4) to a final concentration of 10 μg/mL. For each sample, 100 μL of the substrate in buffer and 15 μL of the enzyme extract were mixed and fluorescence was measured at 485/530 nm. Lipase activity was measured using 4-methylumbelliferyl butyrate (19362; Sigma-Aldrich^®^; Buchs, Switzerland) dissolved in DMSO and then diluted in 200 mM phosphate buffer (pH 7.5) to a final concentration of 0.4 mM. For each sample, 250 μL of the substrate with buffer and 15 μL of the enzyme extract were mixed and fluorescence was measured at 355/460 nm. EnzChek^®^ Ultra Amylase Assay Kit (E33651; Molecular Probes^TM^; Waltham, MA, USA) was used to assess the amylase activity. The fluorescent labeled starch derivate was dissolved in 50 mM sodium acetate buffer (pH 4.0) and then diluted in 50 mM MOPS (pH 6.9) to a final concentration of 200 μg/mL. For each sample, 50 μL of the substrate in buffer and 15 μL of the enzyme extract were mixed and fluorescence was measured at 485/538 nm (excitation/emission) (Cytation 3 Cell Imaging Multi-Mode Reader; Waltham, MA, USA).

All enzyme samples were assayed in duplicate. Enzyme activities were expressed both as relative fluorescence units (RFU) per larva (total activity) and per mg dry body mass.

### 2.4. Statistical Analysis

Data were analyzed for possible outliers using the ROUT method from GraphPad Prism v.6.01 (San Diego, CA, USA). Statistical analyses were performed using SPSS v.23 (Armonk, NY, USA). Data were tested for normality of distribution and homogeneity of variance. To fulfill this, gene expression data were Log_10_-transformed. For each studied variable, significant differences among sampling points were determined using the One-Way Analysis of Variance (ANOVA) followed by the post-hoc Tukey test. Data were considered statistically significant when *p* < 0.05. For visualizing the data, SigmaPlot v.12 (Santa Clara, CA, USA) was used, where they were shown as mean ± standard error of the mean (SEM).

## 3. Results

### 3.1. Growth

Sarasquete et al. [10] divided the early growth of the thick-lipped grey mullet (the same fish stock as the present study) into three different periods. (A) From 5 dph to 22 dph, period of slow growth (growth rate 3.93% per day), (B) from 22 dph to 54 dph, period of fast growth (growth rate 13.45% per day), and (C) from 54 dph to 99 dph, period of intermediate growth (growth rate 6.81% per day) (Figure 1).

### 3.2. Molecular Sequencing

The final sequences for *actb*, *cel*, *pla2g1b*, and *atp4a*, after merging the different fragments (intermediate, 3′-, and/or 5′-ends, if applicable), obtained for each gene consisted of 1367, 1759, 231, and 2285 bp, respectively (GenBank acc. no. MH350431, MH350432, MH350433, and MH350434, respectively). Full-length *beta actin* and *carboxyl ester lipase* nucleotide sequences comprised open reading frames (ORFs) of 1125 and 1665 bp encoding 375 and 555-amino acid (aa) proteins, respectively. Partial *pancreatic phospholipase a2* and gastric H^+^/K^+^-ATPase sequences consisted of ORFs with 231 and 2283 bp coding for 77 and 761-aa proteins, respectively. Blast analyses for all the nucleotide and aa sequences are shown in Supplementary Table S5. Detailed features of the obtained nucleotide and their putative aa sequences are provided in Supplementary Figures S1–S4.

### 3.3. Gene Expression

The reference gene *actb* showed an extremely low variation among all samples, with a cycle threshold (Ct) = 20.40 ± 0.05 (mean ± coefficient of variation). Similarly, comparison of the average Ct value of *actb* of the calibrator sample, which was run in all the plates, showed a negligible inter-assay (black bars lengths) and intra-assay (SEM bars) variations for all the studied genes (Supplementary Figure S5).

**Figure 2:**
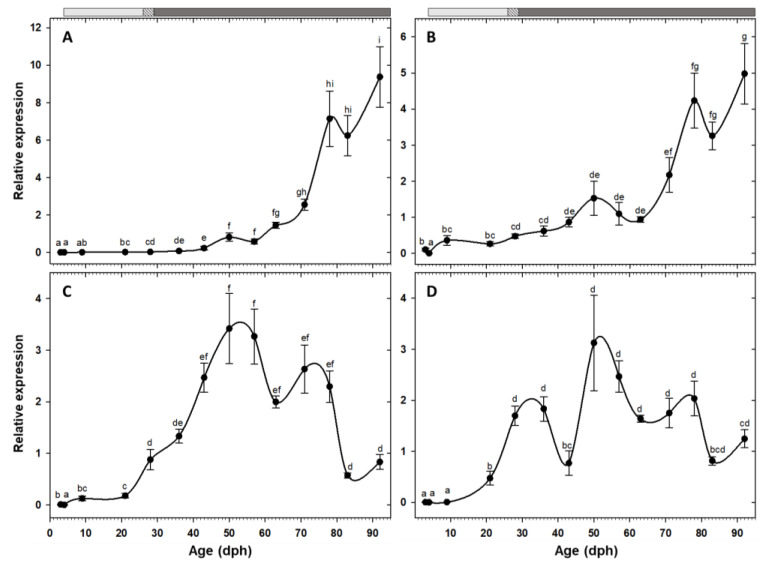
Transcriptional changes of genes related to protein digestion; *try2* (**A**), *ctr* (**B**), *pga2* (**C**), and *atp4a* (**D**) in whole-body *C. labrosus* individuals during development (N = 6 to 10 individuals per sampling point). Different letters denote statistically significant differences between sampling times (*p* < 0.05). Light grey, dashed, and dark grey bars on top of the graph, represent periods with live prey, co-feeding, and inert diet, respectively.

**Figure 3:**
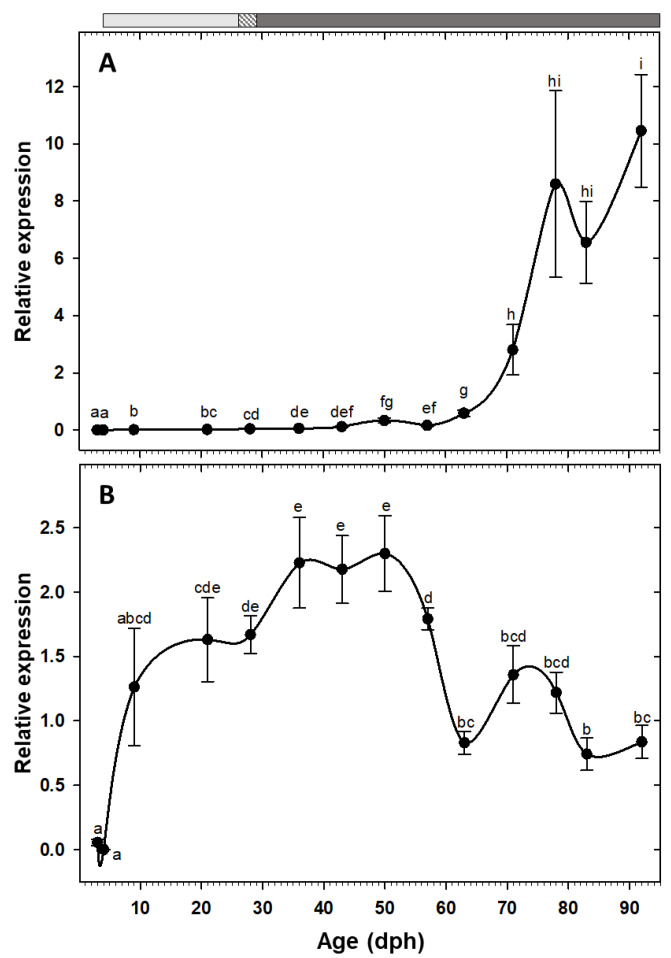
Transcriptional changes of lipases; *cel* (**A**) and *pla2g1b* (**B**) in whole-body *C. labrosus* individuals during development (N = 6 to 10 individuals per sampling point). The rest of legend as in Figure 2.

**Figure 4:**
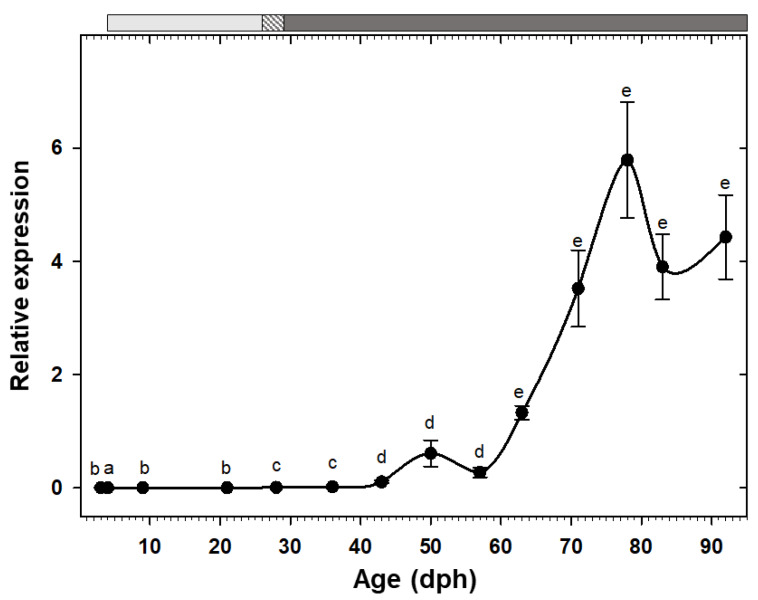
Transcriptional changes of *amy2a* in whole-body *C. labrosus* individuals during development (N = 6 to 10 individuals per sampling point). The rest of legend as in Figure 2.

Expression of the two pancreatic enzymes *try2* and *ctr* had a similar pattern, being low at hatching with a rising trend toward posterior developmental stages and a drastic increase from the 11th week (78 dph) onward (Figure 2A,B). Similarly, the two gastric genes, *pga2* and *atp4a*, also had comparable expression patterns, both increased after the mouth opening and reached to maximum around 50 dph, followed by a stage of relatively constant levels, and a decreasing trend from 83 dph onward (Figure 2C,D). Similar to the other pancreatic enzymes, *cel* expression showed a gradual increasing tendency toward the end of the study period, with a significant up-regulation from 71 dph onward (Figure 3A). Expression of *pla2g1b*, on the other hand, had a completely different pattern, it increased after mouth opening, reached to maximum levels from 21 dph until 57 dph, after which it declined drastically and maintained low levels until the end of the study period (Figure 3B). Expression of *amy2a* showed a similar trend to other pancreatic enzymes, being low during the first weeks of life, increasing gradually toward more advanced developmental stages, followed by a drastic up-regulation from 63 dph onward (Figure 4).

**Figure 5:**
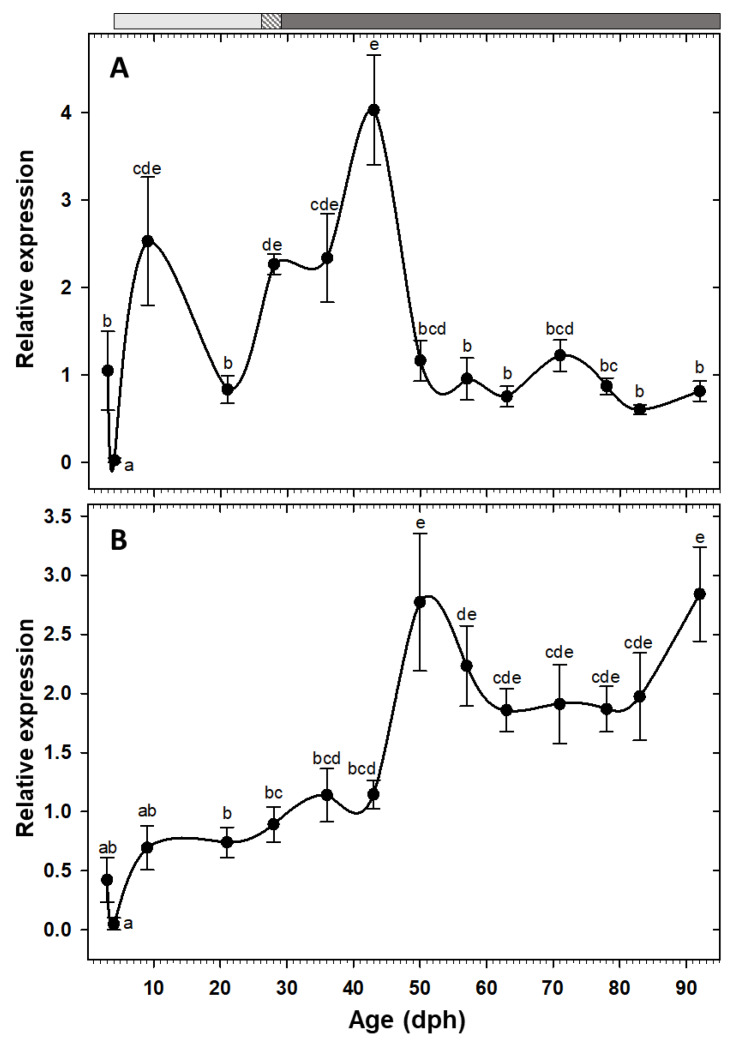
Transcriptional changes of somatotropic factors; *gh* (**A**) and *igf1* (**B**) in whole-body *C. labrosus* individuals during development (N = 6 to 10 individuals per sampling point). The rest of legend as in Figure 2.

The somatotropic factors exhibited opposite patterns (Figure 5). Expression of *gh* increased significantly at 9 dph and maintained the high values, with an exceptional decrease at 21 dph, until reaching to maximum levels at 43 dph. Posteriorly, expression of this gene decreased significantly and maintained relatively low levels during the rest of the study period (Figure 5A). Nevertheless, *igf1* showed a gradual increasing tendency from early days, but it did not reach to the maximum level until 50 dph, beyond which the values were relatively constant until the end of the study (Figure 5B).

### 3.4. Enzyme Activity Analyses

**Figure 6:**
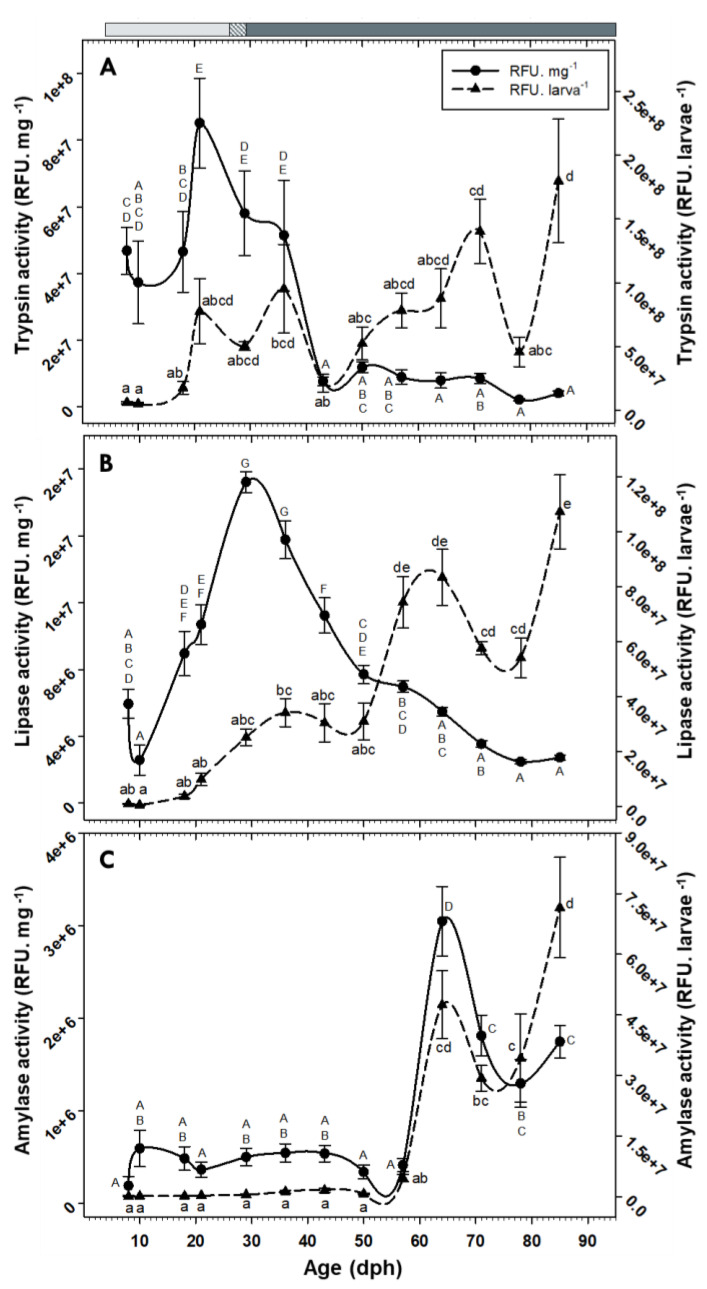
Changes in the activity of trypsin (**A**), lipase (**B**), and amylase (**C**) in *C. labrosus* individuals during development (N = 7 to 13 individuals per sampling point). The rest of legend as in Figure 2.

No pepsin or chymotrypsin activities were detected in individuals of *C. labrosus* at the evaluated ages. In contrast, activities of trypsin and lipase increased over time and exhibited maximum levels in older fish. The activity of these enzymes per mg dry body mass showed a general increasing trend at the beginning of the study (maximum levels at 21 dph and 29 dph, respectively) but dropped significantly afterward (Figure 6A,B). Amylase activity, either per larva or per mg dry body mass, showed low and stable levels until 57 dph but increased drastically from that age onward (Figure 6C).

## 4. Discussion

### 4.1. Growth

As it has been previously described in the same fish stock as the present study [10], the slow growth period (period A) during early days after hatching in this fish stock, has been attributed to prioritizing the development of organs involved in food detection, digestion, and assimilation rather than somatic growth. Fast growth period (period B), on the other hand, has been characterized by a substantial improvement of locomotive function due to its crucial role for food capture and predator avoidance. Finally, intermediate growth rate period (period C) has been considered as a sign to reach to the common isometric profile obtained in juvenile stages. Similar results, three periods of different growth rates, have been obtained during early growth (0–80 dph) of this species under mesocosm conditions [2], although the inflection ages do not exactly match the data in this fish stock [10], probably due to differences in water temperature or rearing conditions, among others, which can substantially modify the development of organs and physiological functions during early stages.

### 4.2. Sequences

The Blast analyses at both nucleotide (blastn) and aa (blastp) levels showed high identity of the sequences of the evaluated proteins (*beta actin*, *bile salt-activated lipase*, *pancreatic phospholipase A2*, and *gastric proton pump)* to their homologues in other teleosts (Supplementary Table S5). Besides, InterPro analysis [22] revealed the fitting of each one of the putative protein sequences into the corresponding Protein family (Supplementary Figures S1–S4). These results clearly validate their use for the ontogeny study of the corresponding transcript regarding their digestive function (see below).

### 4.3. Ontogeny of Digestive Function

#### 4.3.1. Protein Digestion

During the early life stage of marine fish with altricial development, pancreatic proteases are of major importance due to lack of a functional stomach and its acid proteolytic enzymes [23]. In our study, *try2* and *ctr* transcripts were detected before mouth opening (at 3 dph), suggesting that their expression is genetically programmed, rather than being dietary triggered [17,24]. These findings are in agreement with the histological analysis of the fish stock from this experiment, in which rudimentary exocrine pancreas was detected at 3 dph [10].

The drastic increases in expression of *try2* and *ctr* in our study, coincides with the end of the metamorphosis period (23–78 dph) and could imply the maturation of the secreting tissue, exocrine pancreases in this case [10]. Similar findings have been reported in *Solea solea*, a species without a functional stomach, in which alkaline proteases play a major role even in juvenile and adult stages [7,25]. On the contrary, these findings contrast the pattern obtained in a broad range of carnivorous species, in which *trypsinogen* expression has a decreasing trend during ontogeny [5,8,26,27,28].

While there was a general correspondence between *try2* expression and trypsin total activity patterns, trypsin activity per mg dry body mass dropped from 36 dph onward, coinciding roughly with the beginning of the inert diet period, suggesting a putative influence of supplied feed type. The effect of the nature of dietary protein on trypsin activity, and a higher specific activity of this enzyme in *Artemia* fed larvae, compared to the ones fed compound diets with different protein contents, have been previously reported in *Dicentrarchus labrax* [29].

The lack of results when measuring chymotrypsin activity during a long initial developmental period of *C. labrosus*, in spite of the fact that its gene expression was clearly detected, suggest the existence of some kind of post transcriptional/translational regulatory mechanism, which could serve as a preparative system to be used when necessary. Thus, detecting such activity at increasing levels in *C. labrosus* larger individuals, from, 45 g to 329 g [30], indicates that this enzyme/protein may appear later in juvenile/pre-adult stages. In the absence of chymotrypsin, trypsin could be considered as the most important pancreatic proteolytic enzyme in thick-lipped grey mullet at least during the first three month of life, as reported for other fish larvae [23]. Besides, the high activity of trypsin compared to other enzymes during the first 40 days of life indicates that proteins would represent the major energy resource in this period.

The development of a functional stomach is considered a key step in the maturation of the digestive system in fish [23]. It is suggested that *pga2* and *atp4a* expression can be used as a molecular marker for stomach differentiation and performance [31,32]. In some species, morphologic formation of gastric glands and *pga2* expression are simultaneous [26,33]. Correspondingly, in our study an increasing trend of *pga2* and *atp4a* expression from 21 dph onward coincided with proliferation of gastric glands in this species at 23 dph (Figure 1), although maximum expression levels and therefore, expected fully stomach functionality, was not achieved until 50 dph. The decreasing trend measured beyond this point is not in agreement with findings in species with carnivorous habits, in which the mRNA levels of these genes increase toward advanced developmental stages [5,9,17,34].

While *pepsinogen* expression and pepsin specific activity patterns are usually reported to be in line during the ontogeny of fish species [5,9,35], our attempts to detect pepsin activity by using a highly sensitive fluorometric substrate, failed. Similarly, no acid protease activity was detected in the larvae of another mullet species (*Mugil cephalus*) until 79 dph, and the authors suggested a crucial role of alkaline proteases in this family [34]. Thick-lipped grey mullet of larger size (45–329 g), has shown decreasing pepsin activity levels by age, pointing to a minor role of stomach digestion even in adult stages [30]. Therefore, the lack of pepsin detection in this study suggest that despite the availability of anatomically developed gastric glands and genetic machinery, post-transcriptional/translational pathways hamper the functionality of stomach at least until the first three months of life. Absence of pepsin activity despite detectable levels of *pepsinogen* transcripts has been also reported in *Solea senegalensis* juveniles [36]. Consequently, we can suggest that while *pepsinogen* and *gastric proton pump* expression are probably related to the morphologic appearance of gastric glands and structurally developed stomach, the low functionally of this organ can be confirmed by the absence of pepsin activity and/or acidification levels.

Overall, our results regarding protein digestion contrast the usual trend described in marine fish species with carnivorous preference, where the decrease in specific activity of pancreatic proteases with development is compensated by increasing pepsin activity [5,37]. According to histological analysis [10], pinocytotic vesicles of the posterior intestine were evident until the end of the study period (99 dph). These may indicate a crucial role of intracellular protein digestion even at post-larvae and early juvenile stages in this species.

#### 4.3.2. Lipid Digestion

Lipids are considered as key nutrients during early life stages due to the high requirements in energy and structural components of the larvae for growth and development [38]. Detection of *cel* transcripts as early as hatching in some fish species, as in our study, has been attributed to an early potential for lipid digestion [5,39,40]. However, opening of bile and pancreatic ducts into the anterior intestine is not produced until around 5–6 dph [10], this indicating a temporal displacement between the first appearance and its putative functionality. Similar to some other fish species, *cel* expression showed a significant up-regulation at advanced ages in our study, probably due to an age-dependent relationship between *cel* transcriptional levels and differentiation and development of exocrine pancreas and pyloric caeca [17,41,42].

Unlike the high concurrency between *cel* expression and total lipase activity, the activity of this enzyme per mg dry body mass sharply increased during the live prey feeding and decreased steadily along the inert diet period, implying a putative effect of feed type on regulation of lipase activity. Bile salt activated lipase is the only pancreatic neutral lipase in teleosts and has a high efficiency in digestion of triacylglycerols with polyunsaturated fatty acids [41]. Influence of the lipid composition of feeds on modulations of larval lipase activity, as well as the higher specific activity of this enzyme in live prey fed compared to microdiet fed larvae, have been reported in other fish species [43,44,45]. Moreover, lipase activity peak in this study (29 dph) concurred with the full functionality of gall bladder at 25 dph [10]. Likewise, maximum lipase activity in *Seriola rivoliana* has been attributed to bile secretion, which plays an important part in emulsification and neutralization of chyme and a more efficient hydrolysis and absorption processes [37]. In any case, the activity pattern in our study also contrasts to the usual tendency in carnivorous species, where lipase specific activity generally increases during ontogeny [45]. This could indicate a limited role of lipids during post-larval/pre-juvenile stages of *C. labrosus*.

Phospholipase A2 is a group of enzymes that are responsible for digestion of phospholipids in the fish intestine [46]. Phospholipids are essential part of the membrane structure and play an important role in proteins posttranscriptional regulation and as messenger molecules [47]. Besides, they are vital as energy resource and are considered as the most important lipids during the early larval stages. Dietary phospholipid requirement is usually high at the beginning of the fish exogenous feeding and decreases when approaching to the juvenile stage [48]. This could explain the high *pla2g1b* expression during early stages (21–57 dph) and the following decline found in the present study, as previously reported in other species [28].

Pyloric caeca are involved in lipid digestion by increasing the absorption surface area [49]. Accordingly, the maximum expression of *pla2g1b* found in the present study could be related to the histological development of this tissue at 22 dph [10,50]. Besides, *pla2g1b* peak matches the fastest growth period (22–57 dph) during the thick-lipped grey mullet early development (Figure 1), suggesting that this enzyme might be required for an optimum growth performance [27].

The alternation between expression levels of *pla2* and *cel* during ontogeny found in the present study has been described in other fish species, with *pla2* preceeding *cel* in *Labrus bergylta* and *Sparus aurata*, and the opposite in *Gadus morhua* [27,38,42]. This could to be a species-specific indicator of dietary requirements and/or physiological capacities at different development stages. Therefore, we suggest that while *C. labrosus* may require certain amounts of dietary phospholipids during early larval stages, post-larvae and juveniles should depend more on diets rich in wax esters and triacylglycerols of polyunsaturated fatty acids.

#### 4.3.3. Carbohydrate Digestion

Natural diet of most marine fish larvae is rich in lipids and proteins, with lower amounts of carbohydrates [38]. Therefore, the role of amylase in early fish larval stages is still unclear and has been correlated to the digestion of the yolk sac [51] or the intake of phytoplankton [27]. In this study, amylase transcripts were detected from 3 dph (before mouth opening) and displayed a gradual increase and a significant up-regulation at the end of the study. This continuous increasing trend was also observed in other herbivorous/omnivorous fish species [7,52]. However, it contrasts to the results obtained in a wide range of marine species with carnivorous habits, where *amy2a* expression is high during early larval stages and is down-regulated afterward [27,28,42,53,54].

Interestingly, amylase activity both per larva and per mg dry body mass showed a similar pattern; both showed low values for most part of the development but exhibited a dramatic increase from 64 dph onward. This sharp escalation, not only in total secretion but also in synthesis rate of this enzyme over time, is in accordance with results obtained in another mullet species (*M. cephalus*), where amylase specific activity increased significantly at 79 dph [34]. Moreover, the increases in amylase gene expression and activity obtained here cannot be attributed to changes in dietary carbohydrates content, since the same commercial feed was supplied at the mentioned sampling times. Lack of correlation between feeding protocol and amylase expression and/or activity has been previously evidenced in fish species, and the authors suggested a genetically programmed digestive response at this early stage [29,55].

The extent of amylase secretion could be linked to fish feeding habits, with herbivorous and omnivorous fish showing higher α-amylase activity than carnivorous species [52,56]. Therefore, the significant increase in *amy2a* expression and activity highlights the shift of the thick-lipped grey mullet from zooplanktivorous to omnivorous/detrivorous feeding habit, in line with the change from larval to juvenile/adult stages.

This comparatively increasing role of amylase to either trypsin or lipase within the digestive equipment could be a strong support for inclusion of starch or other low cost carbohydrate-based compounds in formulated diets of *C. labrosus* post-larvae/early juveniles, in a similar way to what has been suggested in *M. cephalus* [34], making this species a great candidate for an economically and environmentally sustainable aquaculture.

### 4.4. Ontogeny of gh/igf1 Axis

Fish transition from larvae to juvenile state is characterized by important series of molecular, biochemical, and morphological modifications. Gh and Igf1 play a critical role during this developmental stage [12]. In our study, *gh* transcripts were detected at the first sampling day (3 dph). Early *gh* expression has been previously reported in unfertilized eggs and recently hatched larvae in other fish species [12,13,57], highlighting the crucial role of this hormone during these early days.

In the present work, high *gh* expression during the first weeks was followed by a significant decline, maintaining relatively low levels for the rest of the study period. Similar *gh* expression pattern had been attributed to the impressive fish growth during first weeks after hatching that might be fulfilled by endogenous Gh production [13,58]. Interestingly, high *gh* expression levels in our study strongly corresponds with the fast growth period (22–54 dph) of thick-lipped grey mullet during early development (Figure 1). High correlation between fast somatic growth and elevated *gh* expression levels has been also reported in *Rachycentron canadum* [59].

Expression of *igf1* was relatively low during initial stages and after a peak at 50 dph, it was maintained at high levels during the rest of the sampling points. The significant increase of *igf1* expression in the late larval stages could be linked to numerous processes that are involved in the transition to the juvenile stage [12,13,58]. The fundamental role of Igf1 on growth, differentiation, and morphogenesis has been described in different vertebrates [60,61], including during acclimation process of this species to rearing condition at early life stages (101 dph) [62].

Gh stimulates the growth directly, by DNA proliferation, protein synthesis, and lipolysis in muscle, or indirectly, by inducing the production and release of Igf1 [63,64]. There are solid evidences for the existence of a functional Gh/I/Igf1 axis in adult fish, which is regulated through negative feedback [61]. However, whether such axis is functional, i.e., Gh action is direct or through Igfs, during the fish early development it is unknown [12]. In our study, *igf1* significant up-regulation was followed by an immediate *gh* down-regulation, implying a putative role of *igf1* as a modulator of a negative feedback mechanism to trade-off *gh* transcription and overstimulation. In other words, our findings point out to the establishment of Gh//Igf1 axis from 50 dph onward, at the middle of the metamorphosis period (23–78 dph) [10]. In some other species, on the other hand, Gh//Igf1 axis functionality was started at onset of the exogenous feeding or at the beginning of metamorphosis [58,65].

## 5. Conclusions

Most of the transcriptional and biochemical parameters addressed in this study are in accordance with major histological events during the ontogeny of this species. On the other hand, in spite of previous reports suggesting a precocious intestinal maturation in *C. labrosus* (8–20 dph), our results evidenced that maturation of digestive system and acquisition of adult mode of digestion occurs much later (around 60–70 dph). Additionally, expression patterns of key digestive enzymes and somatotropic factors found in this work were not regulated by the feeding protocol but rather genetically programmed. However, activity pattern of digestive enzymes per mg dry body mass, namely trypsin and lipase, could have been influenced by the offered feed supply. Overall, patterns of digestive enzyme precursors and their activity highlight the omnivorous/detritivorous feeding habit of *C. labrosus* and show an obvious discordance with the usual trend described in carnivorous fish. All these findings support this species as a viable option for a sustainable aquaculture based on the use of low protein-high carbohydrate feeds. Moreover, our results provide valuable information on potentials and capacities of *C. labrosus* at different critical developmental stages that may guide the on-farm feeding practices. Finally, synchronization of fast growth with *gh* up-regulation period in thick-lipped grey mullet underlines the importance of this gene during early development and growth. Furthermore, our results point out to an independent expression of the studied somatotropic genes during the first 40 dph and a functional Gh/Igf1 axis approximately from 50 dph onward.

## Figures and Tables

**Table 1:** Oligonucleotides used for quantitative real-time PCR.

Primer	Direction	Sequence (5′–3′)	Position ^1^	Amplicon Size (bp)	Concentration (nM)	Efficiency	R^2^	Amplification Range	Accession Number
Q-*actb*	Forward	TCTTCCAGCCTTCCTTCCTTG	863	108	200	1.04	0.998	5 ng–5 pg	MH350431
	Reverse	TGTTGGCGTACAGGTCCTTACGG	970						
Q-*atp4a*	Forward	TTGCCTACACGCTAACCAAA	2027	112	200	0.93	0.996	5 ng–5 pg	MH350434
	Reverse	GCCAGTTCGATGAAGAGGAT	2138						
Q-*pla2*	Forward	ACACCTGTTGATGACCTGGA	37	143	200	0.94	0.997	5 ng–5 pg	MH350433
	Reverse	GTCTTGGTGGCCTTGTCAC	179						
Q-*cel*	Forward	CTGACCATGCTGATGACCTG	1360	101	200	1	0.998	5 ng–500 fg	MH350432
	Reverse	GGCAATCATGTAACCGGAGA	1460						
Q-*pga2* ^2^	Forward	AAGATGAAGTGGCTCGTGGTT	-	122	200	0.94	0.999	5 ng–50 pg	KC195968
	Reverse	TCTTCCCACAATCCTTTCTCC	-						
Q-*try2* ^2^	Forward	CTCCAGAACACAGCCATGAAG	-	140	400	0.99	0.999	5 ng–500 fg	KF684940
	Reverse	ACGTTCAGAGAGGCCTGGTAG	-						
Q-*ctr* ^2^	Forward	CGTCCCTTCAGGATTATACCG	-	138	400	0.97	0.998	5 ng–500 fg	KC195969
	Reverse	AGTTGGAGGAACGGTCATGTT	-						
Q-*amy2a* ^2^	Forward	CCAAACTGGGAACTGTCATCAG	-	129	400	0.94	0.996	5 ng–500 fg	KF684941
	Reverse	TCTGGTTGTCGTGGTTGTCA	-						
Q-*gh*	Forward	ATCTTCCCTGACGACTCTGC	-	121	200	1.03	0.998	5 ng–50 pg	KC195966
	Reverse	GGTATGTCTCCACCTTGTGC	-						
Q-*igf1*	Forward	GGAACACACAGGTCAAACGA	-	132	200	1	0.995	5 ng–5 pg	KC195967
	Reverse	CGCTCCCTTTCTCATAGTTG	-						

^1^ Positions are relative to *C. labrosus* cDNA sequences obtained in this study; ^2^ From Pujante et al. [20]; R^2^: Coefficient of Determination.

## Supplementary Materials

The following are available online at https://www.mdpi.com/2076-2615/10/5/874/s1, Figure S1: Nucleotide and amino acid sequences for *C. labrosus* carboxyl ester lipase, Figure S2: Nucleotide and amino acid sequences for *C. labrosus* pancreatic phospholipase A2, Figure S3: Nucleotide and amino acid sequences for *C. labrosus* gastric proton pump, Figure S4: Nucleotide and amino acid sequences for *C. labrosus* β-actin, Figure S5: Comparison of cycle threshold (Ct) average values of the calibrator sample for all the studied genes, Table S1: Nutritional value of the commercial feeds used in the intensive culture of *C. labrosus*, Table S2: Sampling points of *C. labrosus* in relation to ontogenic events, according to the key ontogenic events defined by histochemistry approaches [10], Table S3: Oligonucleotides used for cloning *C. labrosus* cDNA sequences, Table S4: Teleost species and GenBank accession numbers of cDNA sequences used to design degenerate primers., Table S5: Top five blast hits for the cloned cDNAs and their putative amino acid translations from *C. labrosus*.

## Abbreviations

*actb*	*actin beta*
*amy2a*	*pancreatic alpha amylase*
*atp4a*	*gastric H^+^/K^+^-ATPase*
*cel*	*carboxyl ester lipase precursor*
*ctr*	*chymotrypsinogen precursor*
*gh*	*growth hormone*
*igf1*	*insulin-like growth factor 1*
*pga2*	*pepsinogen 2 precursor*
*pla2g1b*	*pancreatic phospholipase A2*
*try2*	*trypsinogen 2 precursor*
aa	amino acid
Ct	cycle threshold
dph	days post hatch
Gh	growth hormone
GIT	gastrointestinal tract
Igf1	insulin-like growth factor 1
ORF	open reading frame
qPCR	quantitative real-time PCR
SEM	standard error of the mean
