# Supplementary material for: Ontogeny of Expression and Activity of Digestive Enzymes and Establishment of gh/igf1 Axis in the Omnivorous Fish Chelon labrosus
Source: Animals (Basel). 2020 May 18;10(5):874. doi: 10.3390/ani10050874 (PMC7278486; doi:10.3390/ani10050874)
Supplement: Supplementary file 1 [file animals-10-00874-s001.pdf]

animals

MDPI

Supplementary Material

Ontogeny of expression and activity of digestive enzymes and establishment of *gh/igf1* axis in the omnivorous fish *Chelon labrosus*

Neda Gilannejad [1,*], Verónica de las Heras [1,†], Juan Antonio Martos-Sitcha [2], Francisco J. Moyano [3], Manuel Yúfera [1] and Gonzalo Martínez-Rodríguez [1]

1. Instituto de Ciencias Marinas de Andalucía (ICMAN-CSIC), 11519 Puerto Real, Cádiz, Spain; veronica.delasher@alum.uca.es (V.d.l.H.); manuel.yufera@icman.csic.es (M.Y.); gonzalo.martinez@csic.es (G.M.-R.)
2. Department of Biology, Faculty of Marine and Environmental Sciences, Instituto Universitario de Investigación Marina (INMAR), Campus de Excelencia Internacional del Mar (CEI-MAR), University of Cádiz, 11519 Puerto Real, Cádiz, Spain; juanantonio.sitcha@uca.es
3. Departamento de Biología y Geología, Facultad de Ciencias, Campus de Excelencia Internacional del Mar (CEI-MAR), Universidad de Almería, La Cañada de San Urbano, 04120 Almería, Spain; fjmoyano@ual.es

* Correspondence: neda.gilannejad@icman.csic.es

† Current address: Futuna Blue España S.L., Dársena Comercial Pesquera s/n, 11500 El Puerto de Santa María, Cádiz, Spain.

Table S1. Nutritional value of the commercial feeds used in the intensive culture of *C. labrosus* (Skretting, Burgos, Spain).

	Pellet (0.1 mm)	Pellet (0.2 mm)	Pellet (0.5 mm)
Protein (%)	60	60	58
Lipid (%)	15	15	17
Ash (%)	9	9	10
Fiber (%)	0.5	0.5	0.6
Total P (%)	1.5	1.5	1.3
Energy Density (MJ/kg)	19.3	19.3	19.9

Table S2. Sampling points of *C. labrosus* in relation to ontogenic events, according to the key ontogenic events defined by histochemistry approaches [10].

Age (dph)	Purpose	Ontogeny event
3	Expression	Before mouth opening
4	Expression	Mouth opening
8	Activity	Period of slow growth (Growth rate 3.93 % per day)
9	Expression	
10	Activity	
18	Activity	
21	Expression + Activity	
28	Expression	Period of fast growth (Growth rate 13.45 % per day)
29	Activity	
36	Expression + Activity	
43	Expression + Activity	
50	Expression + Activity	
57	Expression + Activity	Period of medium growth (Growth rate 6.81 % per day)
63	Expression	
64	Activity	

71	Expression + Activity
78	Expression + Activity
83	Expression
85	Activity
92	Expression

Table S3. Oligonucleotides used for cloning *C. labrosus* cDNA sequences.

	Primer	Direction	Sequence (5′-3′)	Position [1]
Intermediate amplification	apATP4A-F1	Forward	CARTGYCTBATGTGGGTBGC	1
	apATP4A-F2	Forward	YATCTGCYTCATYGCCTTYG	27
	apATP4A-R1	Reverse	TCSGGBACRTARGCYACCAC	701
	clPLA2-F1	Forward	RAYTAYGGMTGCTAYTGYGG	1
	clPLA2-R1	Reverse	TTSYKGTCRCACTCRCAGATRAA	231 + 5
	clCEL-5F3	Forward	YAACTATCTGTACRRYGGYSAGGA	416
	clCEL-5F4	Forward	GACTTCSTVCCNGATSADCCW	930
	clCEL-3R4	Reverse	GCDAAGTTDGYCCAGTAKGCRAT	1477
5′ end amplification	M13 Reverse	Forward	CAGGAAACAGCTATGAC	-
	qRT-actb –Rv [2]	Reverse	GAGCGTAGCCCTCGTAGATG	592
	M13 Reverse	Forward	CAGGAAACAGCTATGAC	-
	T3	Forward	ATTAACCCTCACTAAAG	-
	clBAL5race-R1	Reverse	ACCACCAAATGAGCGAATGT	602
	clBAL5race-R2	Reverse	CAGAGTGCCCACACGGTACC	497
3′ elongation	clATP4A3race-F1	Forward	GCCATGATCTTCTTCATGGC	658
	apATP4A.elongate-R2 [3]	Reverse	GACTGRATGGCDCCDATCTG	2266
	apATP4A.elongate-R3 [3]	Reverse	AAKATVAGDCGDCCCTGCTC	1987
	apATP4A.elongate-R4 [3]	Reverse	GASCCMGCRATVCCCATGGC	1897
3′ end amplification	qRT-actb-Fw [2]	Forward	CAGGGAGAAGATGACCCAGA	426
	M13 Forward (-20)	Reverse	GTAAAACGACGGCCAG	-
	clCEL3race-F2	Forward	TGGTGGACACCCCTATCGAG	1057
	T7	Reverse	TAATACGACTCACTATAGGG	-

[1] Positions are relative to *C. labrosus* cDNA sequences obtained in this study; [2] From Pujante et al. [18]; [3] From Gilannejad et al. [17].

Table S4. Teleost species and GenBank accession numbers of cDNA sequences used to design degenerate primers.

cel	***pla2g1b***
Epinephelus coioides (EU683730)	*Dicentrarchus labrax* (AJ132762)
Thunnus orientalis (AB859993) (AB859992)	*Neolamprologus brichardi* (XM_006805419)
Takifugu rubripes (XM_003978374),	*Haplochromis burtoni* (XM_005933279)
Pseudopleuronectes americanus (AF512561)	*Pundamilia nyererei* (XM_005733538)
Sparus aurata (JX975714),	*Maylandia zebra* (XM_004558519)
Salmo salar (L23929)	*Oryzias latipes* (XM_004086573)
	Oreochromis niloticus (XM_003445672)
	Gadus morhua (EX726814)
	Pagrus major (AB009286)
	Astyanax mexicanus (XM_007241732)
	Takifugu rubripes (XM_003976624)

Table S5. Top five blast hits for the cloned cDNAs and their putative amino acid translations from *C. labrosus*, with descriptions of percentage of query cover, E value, percentage of identity and similarity, and accession numbers. (-NA-: Not Applicable).

***gene*/Protein**	**Description**	**Query cover**	**E value**	**Identity**	**Similarity**	**Acc. number**
beta actin	PREDICTED: *Seriola lalandi dorsalis* actin, cytoplasmic 1 (LOC111647584), mRNA	98 %	0.0	97 %	-NA-	XM_023397206.1
	PREDICTED: *Lates calcarifer* actin beta (*actb*), mRNA	99 %	0.0	97 %	-NA-	XM_018667666.1
	PREDICTED: *Seriola dumerili* actin beta (*actb*), mRNA	98 %	0.0	97 %	-NA-	XM_022757055.1
	Epinephelus coioides beta actin mRNA, complete cds	98 %	0.0	97 %	-NA-	AY510710.2
	Sillago japonica mRNA for actin, complete cds	98 %	0.0	97 %	-NA-	AB288085.1
Beta actin	PREDICTED: actin, beta isoform X1 [*Cynoglossus semilaevis*]	100 %	0.0	100 %	100 %	XP_008328442.1
	actin, beta [*Cynoglossus semilaevis*]	100 %	0.0	100 %	100 %	NP_001295108.1
	beta actin [*Acanthopagrus schlegelii*]	100 %	0.0	99 %	100 %	AAR84618.1
	actin, cytoplasmic 1 isoform X1 [*Oncorhynchus mykiss*]	100 %	0.0	99 %	100 %	XP_021451454.1
	actin, cytoplasmic 1 [*Acanthochromis polyacanthus*]	100 %	0.0	99 %	100 %	XP_022063516.1
bile salt-activated lipase	PREDICTED: *Stegastes partitus* bile salt-activated lipase-like (LOC103362077), mRNA	94 %	0.0	84 %	-NA-	XM_008288338.1
	Dicentrarchus labrax bile salt-activated lipase (CEL) mRNA, complete cds	95 %	0.0	83 %	-NA-	KF857291.1
	PREDICTED: *Larimichthys crocea* bile salt-activated lipase-like (LOC109143108), mRNA	94 %	0.0	83 %	-NA-	XM_019279260.1
	PREDICTED: *Amphiprion ocellaris* bile salt-activated lipase-like (LOC111571549), mRNA	93 %	0.0	83 %	-NA-	XM_023274768.1
	PREDICTED: *Stegastes partitus* bile salt-activated lipase-like (LOC103362076), mRNA	93 %	0.0	83 %	-NA-	XM_008288337.1
Bile salt-activated lipase	bile salt-activated lipase [*Dicentrarchus labrax*]	100 %	0.0	83 %	91 %	AIT82969.1
	PREDICTED: bile salt-activated lipase-like [*Stegastes partitus*]	100 %	0.0	82 %	92 %	XP_008286560.1

	PREDICTED: bile salt-activated lipase-like [*Lates calcarifer*]	99 %	0.0	83 %	91 %	XP_018548618.1
	bile salt-activated lipase-like [*Amphiprion ocellaris*]	99 %	0.0	82 %	91 %	XP_023130536.1
	bile salt-activated lipase-like [*Seriola dumerili*]	100 %	0.0	82 %	91 %	XP_022623760.1
phospholipase A2	PREDICTED: *Lates calcarifer* phospholipase A2, minor isoenzyme-like (LOC108878618), mRNA	99 %	7e-61	86 %	-NA-	XM_018669495.1
	PREDICTED: *Neolamprologus brichardi* phospholipase A2-like (LOC102782690), mRNA	98 %	2e-56	85%	-NA-	XM_006805419.1
	PREDICTED: *Maylandia zebra* phospholipase A2 group IB (*pla2g1b*), mRNA	98 %	9e-55	84 %	-NA-	XM_004558519.4
	PREDICTED: *Pundamilia nyererei* phospholipase A2, group IB (pancreas) (*pla2g1b*), mRNA	98 %	9e-55	84 %	-NA-	XM_005733538.1
	PREDICTED: *Seriola dumerili* phospholipase A2 group IB (*pla2g1b*), mRNA	99 %	3e-54	84 %	-NA-	XM_022753451.1
Phospholipase A2	PREDICTED: phospholipase A2, minor isoenzyme-like [*Lates calcarifer*]	100 %	1e-40	83 %	93 %	XP_018525011.1
	PREDICTED: phospholipase A2 [*Oreochromis niloticus*]	100 %	8e-40	83 %	9 1%	XP_003445720.1
	PREDICTED: phospholipase A2, minor isoenzyme [*Cynoglossus semilaevis*]	100 %	9e-40	81 %	92 %	XP_008334840.1
	PREDICTED: phospholipase A2-like [*Neolamprologus brichardi*]	100 %	1e-39	83 %	91 %	XP_006805482.1
	phospholipase A2-like [*Acanthochromis polyacanthus*]	100 %	1e-39	81 %	88 %	XP_022071361.1
gastric H+/K+ ATPase alpha subunit	PREDICTED: *Lates calcarifer* ATPase H+/K+ transporting alpha subunit (*atp4a*), mRNA	100 %	0.0	90 %	-NA-	XM_018689652.1
	Siniperca scherzeri proton pump alpha subunit mRNA, complete cds	100 %	0.0	90 %	-NA-	FJ480418.1
	PREDICTED: *Amphiprion ocellaris* ATPase H+/K+ transporting alpha subunit (*atp4a*), mRNA	100 %	0.0	89 %	-NA-	XM_023277150.1
	Siniperca chuatsi gastric H+/K+ ATPase alpha subunit mRNA, complete cds	99 %	0.0	90 %	-NA-	HM165261.1
	PREDICTED: *Acanthochromis polyacanthus* ATPase H+/K+ transporting alpha subunit (*atp4a*), mRNA	100 %	0.0	89 %	-NA-	XM_022196744.1

Gastric H+/K+ ATPase alpha subunit	potassium-transporting ATPase alpha chain 1 [*Acanthochromis polyacanthus*]	100 %	0.0	96 %	98 %	XP_022052436.1
	potassium-transporting ATPase alpha chain 1 [*Amphiprion ocellaris*]	100 %	0.0	96 %	98 %	XP_023132918.1
	PREDICTED: potassium-transporting ATPase alpha chain 1 [*Stegastes partitus*]	100 %	0.0	96 %	98 %	XP_008275275.1
	PREDICTED: potassium-transporting ATPase alpha chain 1 [*Paralichthys olivaceus*]	100 %	0.0	96 %	98%	XP_019948166.1
	PREDICTED: potassium-transporting ATPase alpha chain 1 [*Lates calcarifer*]	100 %	0.0	96 %	98 %	XP_018545168.1

```
      CGATGGCGAAGCTGGGGATTTTGGTTGCCATTGCCGTGTTTCTGGAGACGGTCTCTGCCA 60
   1    M  A  K  L  G  I  L  V  A  I  A  V  F  L  E  T  V  S  A  T

      CCTCTCTTGGTGTCGTGAACACGGAGGGAGGGGCAGTGCAGGGTAAAAACATTTGGCTTG 120
  21    S  L  G  V  V  N  T  E  G  G  A  V  Q  G  K  N  I  W  L  G

      GGTTATTCCGTAGCATGGACGTTTTCAAGGGGATTCCCTTTGCTGACATCCCAGGGAGGT 180
  41    L  F  R  S  M  D  V  F  K  G  I  P  F  A  D  I  P  G  R  F

      TTGAGAAACCAAAGCGTCACCCTGGATGGGATGGTACTTTGAAGGCCACTGACTATAGAC 240
  61    E  K  P  K  R  H  P  G  W  D  G  T  L  K  A  T  D  Y  R  P

      CGAGATGTCTTCAGCTGAACATTCTCATGACTGACACCATGGGCAGTGAGGACTGTCTTT 300
  81    R  C  L  Q  L  N  I  L  M  T  D  T  M  G  S [E  D  C  L  Y]

      ACCTCAACATCTGGGTTCCCCATGGCAGCTCAGTGTCCCGTGATCTACCTGTCATGGTCT 360
 101  [ L  N  I  W  V  P] H  G  S  S  V  S  R  D  L  P  V  M  V  W

                                                             clCEL
      GGATTTATGGTGGAGGATTCCTGGTTGGGGGCGCCATGGGTGCTAACTTCCTGGATAACT 420
 121    I  Y  G  G  G  F  L  V  G  G  A  M  G  A  N  F  L  D  N  Y
              *  *  *

      -5F3 ------------>                                         <---
      ATCTGTACGATGGGCAGGAGATCGCAGACAGAGGCAATGTTATTGTGGTGACGCTGGGGT 480
 141    L  Y  D  G  Q  E  I  A  D  R  G  N  V  I  V  V  T  L  G  Y

     ---- clBAL5race-R2
      ACCGTGTGGGCACTCTGGGATTCCTGAGCACTGGAGACTCCAGTTTGCCTGGAAACTATG 540
 161    R  V  G  T  L  G  F  L  S  T  G  D  S  S  L  P  G  N  Y  G

                                                <------ clBAL5race
      GTCTCTGGGACCAGCAGGCTGCCATTGCGTGGGTGCACAGAAACATTCGCTCATTTGGTG 600
 181    L  W  D  Q  Q  A  A  I  A  W  V  H  R  N  I  R  S [F  G  G]

      -R1
      GTGACCCTGACAACATCACCGTCTTTGGTGAATCTGCAGGTGCAGCCAGTGTTAGCTTCC 660
 201  [ D  P  D  N  I  T  V  F  G  E  S  A  G] A  A  S  V  S  F  Q
                                       *  *  *     *
                                          ▲
      AGACTATTACCCCCCACAACAAAGGGCTGATCAGAAGAGCCATTTCCCAGAGTGGGGTCG 720
 221    T  I  T  P  H  N  K  G  L  I  R  R  A  I  S  Q  S  G  V  A

      CCCTTTGCCCCTGGGCTGTCAACCCTAACCCCCGTAGGTTTGCTGAGGAGATTGCTCTAA 780
 241    L  C  P  W  A  V  N  P  N  P  R  R  F  A  E  E  I  A  L  K

      AGGTCAACTGCCCCACTGATCAAAATATGGCTGCCTGTTTGAAGATGACTGATCCGGGAC 840
 261    V  N  C  P  T  D  Q  N  M  A  A  C  L  K  M  T  D  P  G  L

      TTCTAACAATGGCGGGCACTATCAGTCTGTCCAGCTCTCCTGATAACCCTGTTGTTTTCA 900
 281    L  T  M  A  G  T  I  S  L  S  S  S  P  D  N  P  V  V  F  N

                                     clCEL-5F4 -------->
      ACCTCCTCCTGGCTGCTGTGGTTGACGGAGACTTCCTGCCGGATGAGCCTCAGAATTTGT 960
 301    L  L  L  A  A  V  V  D  G  D  F  L  P  D  E  P  Q  N  L  F

      TCCACAATGCAGCAAGCATTGACTACATAGCTGGAGTCAATGACATGGATGGACATTTGT 1020
 321    H  N  A  A  S  I  D  Y  I  A  G  V  N  D  M  D  G  H  L  F
                                                        ▲
                                         clCEL3race-F2 ------->
      TCACTGCTGTAGATGTTCCAACAGTTAACTCTCCAATGGTGGACACCCCTATCGAGGATG 1080
 341    T  A  V  D  V  P  T  V  N  S  P  M  V  D  T  P  I  E  D  V

      TGAGGAGGCTCTTGACTGCCTACACAAGAGAGAAGGGCAAGGCTGGTGCAGACAACGCCT 1140
 361    R  R  L  L  T  A  Y  T  R  E  K  G  K  A  G  A  D  N  A  Y
              *           *  *
      ACAGCACATACACTTCAACTTGGGGGTCAAGTCCCAGCTGGGAGACCATCAAGAAGACGA 1200
 381    S  T  Y  T  S  T  W  G  S  S  P  S  W  E  T  I  K  K  T  I

      TTGTGGAAATTGGAACAGATTACATCTTCCTGGTTCCTACACAGGCAGCTCTTTATCTTC 1260
 401    V  E  I  G  T  D  Y  I  F  L  V  P  T  Q  A  A  L  Y  L  H
                 *
      ACGCTGGCAGCGGCGCAACCGGACGCACCTACTCTTATCTCTTCTCTGAGCCAAACCGTA 1320
 421    A  G  S  G  A  T  G  R  T  Y  S  Y  L  F  S  E  P  N  R  M

                                                Q-CEL3-F --------->
      TGGGCGGCGTTGCCAGGCCCTACCCCAGCTGGATGGGAGCTGACCATGCTGATGACCTGC 1380
 441    G  G  V  A  R  P  Y  P  S  W  M  G  A  D  H  A  D  D  L  Q
                                                 ▲  *        *
      AATACGTGTTTGGAAAGCCATTCGCCACACCTCTGGGATACTGGCCTCGCCACCGTGACG 1440
 461    Y  V  F  G  K  P  F  A  T  P  L  G  Y  W  P  R  H  R  D  V
```

```
                 <-------------- clCEL-3R4
    <------------ Q-CEL3-R                                  Q-CEL4-F
    TCTCCGGTTACATGATTGCCTACTGGACCAACTTTGCCAAAACTGGTGACCCCAACAAAG 1500
481  S  G  Y  M  I  A  Y  W  T  N  F  A  K  T  G  D  P  N  K  G

    ------------>
    GAGACCTGAGCGTGCCTGCTGTCTGGCCCGAATTCACCGCCTCTGGGCATCAGTTCTTGG 1560
501  D  L  S  V  P  A  V  W  P  E  F  T  A  S  G  H  Q  F  L  E

                                     <----------- Q-CEL4-R
    AGATCAATTCTGATATGAACAGCAACTTTGTGAAGCAGAAGATGAGGATGCGTTACGTGC 1620
521  I  N  S  D  M  N  S  N  F  V  K  Q  K  M  R  M  R  Y  V  H

    ATTTCTGGACCAGCATCCTGCCCAGCCTTCCCACACTCTACTCAGAATAAAGACCTTGTG 1680
541  F  W  T  S  I  L  P  S  L  P  T  L  Y  S  E

    CAACCACATGTGCACCTGCTTTGTGGACCTGTCTAAAGCATTGGTTCATGCAAAATAAAT 1740
    GGATGGCGCATACAAACAT Poly-A 1759
```

Figure S1. Nucleotide (numbered to the right) and amino acid (numbered to the left) sequences for *C. labrosus* carboxyl ester lipase (GenBank acc. no. MH350432). Start and Stop codons are indicated in bold and underlined. All the primers are underlined (except for qPCR oligonucleotides, which are in bold and double underlined) and arrows represent their directions. The Poly-A signal AATAAA is underlined with a thicker line. The UTRs are indicated in gray. The conserved site IPR019819 for Carboxylesterase type-B signature 2 (Prosite PS00941 CARBOXYLESTERASE_B_2; [EDA]-[DG]-C-L-[YTF]-[LIVT]-[DNS]-[LIV]-[LIVFYW]-x-[PQR]) is boxed with a single line. The active site IPR019826 for Carboxylesterase type-B serine active site (Prosite PS00122 CARBOXYLESTERASE_B_1; F-[GR]-G-x(4)-[LIVM]-x-[LIV]-x-G-x-S-[STAG]-G) is boxed with a double line. The amino acids from the conserved protein domain family cd00312 (Esterase_lipase), related to the Feature 1 corresponding with the catalytic triad (active site), are labeled with a solid up arrowhead (▲) under the corresponding amino acids (S^{211}, D^{336}, H^{455}), whereas the related to the Feature 2, corresponding with the substrate binding pocket (chemical binding site), are labeled with an asterisk (*) under the corresponding amino acids (G^{123}, G^{124}, G^{125}, E^{210}, S^{211}, A^{212}, A^{215}, L^{363}, Y^{367}, T^{368}, G^{404}, A^{456}, L^{459}).

```
   clPLA2-F1 ──────→                                Q-PLA2-F ──────────→
   AACTATGGATGCTATTGCGGGCTGGGAGGCTCTGGTACACCTGTTGATGACCTGGACAGA 60
 1 N  Y  G  C  Y  C  G  L  G  G  S  G  T  P  V  D  D  L  D  R
                    ▲
              *     *     *
   TGCTGTCAGGTGCACGACCAGTGCTACAGTGATGCCATGCAGCACAGTGAATGCTGGCCC 120
21 C  C  Q  V  H  D  Q  C  Y  S  D  A  M  Q  H  S  E  C  W  P
             ▲  ▲        ▲
                *                          ←──────────── Q-PLA2-R
   ATTCTGGACAATCCATATACTGAAATATACAGCTTCAGCTGTGACAAGGCCACCAAGACC 180
41 I  L  D  N  P  Y  T  E  I  Y  S  F  S  C  D  K  A  T  K  T
                              ▲
                                                 ←──────── clPLA2-R1
   GTCACCTGTCATAGCAAAGACACTTGTGAGAAGTTCATCTGCGAGTGCGAC 231
61 V  T  C  H  S  K  D  T  C  E  K  F  I  C  E  C  D
                                                   ▲
```

Figure 2. Nucleotide (numbered to the right) and amino acid (numbered to the left) partial sequences for *C. labrosus* pancreatic phospholipase A2 (GenBank acc. no. MH350433). All the primers are underlined (except for qPCR oligonucleotides, which are in bold and double underlined) and arrows represent their directions. The conserved sites IPR001211 for Phospholipase A2 Protein family signature (3 out of 5 motifs) (PRINTS PR00389 PHPHLIPASEA2 motifs 3, 4 and partial 5) are boxed with a single line. The conserved site IPR0033113 for Phospholipase A2, histidine active site (PROSITE pattern PS00118: C-C-{P}-x-H-{LGY}-x-C) is highlighted in gray. The amino acids from the conserved protein domain family cd00125 (PLA2c), related to the Feature 1 corresponding with the catalytic network (active site), are labeled with a solid up arrowhead (▲) under the corresponding amino acids (G^7, H^{25}, D^{26}, Y^{29}, Y^{50}, D^{77}), whereas the related to the Feature 2, corresponding with the primary metal binding site are labeled with an asterisk (*) under the corresponding amino acids (Y^5, G^7, G^9, D^{26}).

```
    apATP4A-F1 ------->      apATP4A-F2 ------->
    CAGTGCCTTATGTGGGTGGCAGCCGCCATCTGTTTCATCGCCTTTGGAATTGAACTTGCA 60
  1 Q  C  L  M  W  V  A  A  A  I  C  F  I  A  F  G  I  E  L  A
    AGAGGGAACCTTACCAGCTTTGATGATCTGTACTTGGCCATCACCCTAATTGCTGTTGTC 120
 21 R  G  N  L  T  S  F  D  D  L  Y  L  A  I  T  L  I  A  V  V
    GTGGTAACTGGTTGCTTTGGTTACTACCAAGAATTCAAAAGCACCAACATCATTGCCAGC 180
 41 V  V  T  G  C  F  G  Y  Y  Q  E  F  K  S  T  N  I  I  A  S
    TTCAAGAATCTGGTGCCACAACAAGCCATGGTGATCCGTGATGGCCAGAAGAACCAGATC 240
 61 F  K  N  L  V  P  Q  Q  A  M  V  I  R  D  G  Q  K  N  Q  I
    AATGCCTACCAGCTCGTGGTGGGGGATTTGGTGGAGATCAAAGGAGGAGACAGAGTCCCC 300
 81 N  A  Y  Q  L  V  V  G  D  L  V  E  I  K  G  G  D  R  V  P
    GCTGACATTCGCATCATCACCGCTCAGAGCTGCAAGGTTGATAACTCATCCCTGACTGGA 360
101 A  D  I  R  I  I  T  A  Q  S  C  K  V  D  N  S  S  L  T  G
    GAGTCTGAACCACAAACCAGGAGCCCCGAATGTACTCATGAAAACCCATTGGAGACCAGA 420
121 E  S  E  P  Q  T  R  S  P  E  C  T  H  E  N  P  L  E  T  R
    AACATTGCATTCTTCTCTACAACCTGTCTGGAAGGTGTGGCCACTGGTATAATCATTAAC 480
141 N  I  A  F  F  S  T  T  C  L  E  G  V  A  T  G  I  I  I  N
    ACAGGCGACCGCACCATCATCGGTCGCATTGCCAGCTTGGCGAGCGGCGTCGGCAACGAG 540
161 T  G  D  R  T  I  I  G  R  I  A  S  L  A  S  G  V  G  N  E
    AAGACACCCATTGCCATAGAAATTGAGCACTTTGTGGACATCATCGCTGGCCTTGCGATC 600
181 K  T  P  I  A  I  E  I  E  H  F  V  D  I  I  A  G  L  A  I
                                                   clATP4A3race-F1
    TTTTTCGGGTTCACATTCTTCGTGGTTGCCATGTTTATCGGATATGCATTCCTGGAAGCC 660
201 F  F  G  F  T  F  F  V  V  A  M  F  I  G  Y  A  F  L  E  A
    ----------------->    <--------- apATP4A-R1
    ATGATCTTCTTCATGGCTATTGTGGTGGCTTATGTGCCAGAAGGACTACTCGCTACAGTT 720
221 M  I  F  F  M  A  I  V  V  A  Y  V  P  E  G  L  L  A  T  V
                                            ▼
                           V        V     V
    ACTGTGTGTTTGTCTCTGACTGCTAAACGACTTGCCAGGAAGAACTGTGTTGTGAAGAAC 780
241 T  V  C  L  S  L  T  A  K  R  L  A  R  K  N  C  V  V  K  N
    CTGGAGGCCGTGGAGACTTTAGGCTCCACGTCGGTGATCTGTTCGGATAAGACGGGCACA 840
261 L  E  A  V  E  T  L  G  S  T  S  V  I  C  S  D  K  T  G  T
                                                 *  *  *
    CTGACCCAGAACAGGATGACTGTAGCTCACCTTTGGTTTGACAACAAGATTCATGCCGCT 900
281 L  T  Q  N  R  M  T  V  A  H  L  W  F  D  N  K  I  H  A  A
    GATACCACTGAAGATCAGTCAGGTCAGAGCTTTGACCAGTCGTCAGAGACGTGGCGCTCT 960
301 D  T  T  E  D  Q  S  G  Q  S  F  D  Q  S  S  E  T  W  R  S
    CTGGCCAGAGTAGCATCCCTGTGCAACAGAGCTGTATTCAGACCCGATCAGGAAGGAGTA 1020
321 L  A  R  V  A  S  L  C  N  R  A  V  F  R  P  D  Q  E  G  V
    CCCATTCCTAAGCGGCTCGTGGTGGGGGATGCATCTGAGACAGCTCTACTGAAGTTCACT 1080
341 P  I  P  K  R  L  V  V  G  D  A  S  E  T  A  L  L  K  F  T
                               *        *
    GAGCTCACTGTAGGGAACATCATAGACTACCGTAATCGCTTCAAGAAAGTGACGGAAGTG 1140
361 E  L  T  V  G  N  I  I  D  Y  R  N  R  F  K  K  V  T  E  V
    CCCTTCAACTCTACCAACAAGTTCCAGCTGTCCATCCATGAACTGGAGGATCCATTGGAC 1200
381 P  F  N  S  T  N  K  F  Q  L  S  I  H  E  L  E  D  P  L  D
    *  *     *        *     *
    CTGCGCTACCTGCTGGTGATGAAGGGAGCCCCGGAGCGAATCTTAGAACGCTGCTCCACC 1260
401 L  R  Y  L  L  V  M  K  G  A  P  E  R  I  L  E  R  C  S  T
                         *  *  *
    ATTTTGATAAAAGGCCAGGAGCTGCCTCTGGATGAGCAGTGGAAAGAGTCTTTCCAGACG 1320
421 I  L  I  K  G  Q  E  L  P  L  D  E  Q  W  K  E  S  F  Q  T
    GCTTACATGGACCTGGGAGGACTGGGAGAGAGAGTACTGGGTTTCTGCCACCTCTACCTC 1380
441 A  Y  M  D  L  G  G  L  G  E  R  V  L  G  F  C  H  L  Y  L
                                  *  *  *
    AATGAGAAAGAGTATCCACGTGGCTTTCAATTTGACGCCGATGAGATGAATTTCACCACG 1440
461 N  E  K  E  Y  P  R  G  F  Q  F  D  A  D  E  M  N  F  T  T
```

```
    TCAGGATTGTGCTTCGCTGGTCTCATCGCAATGATTGACCCTCCAAGAGCCACTGTACCC 1500
481 S  G  L  C  F  A  G  L  I  A  M  I  D  P  P  R  A  T  V  P

    GACGCAGTGATGAAGTGCCGCACTGCTGGTATCAGGGTTGTCATGGTGACCGGCGATCAT 1560
501 D  A  V  M  K  C  R  T  A  G  I  R  V  V  M  V  T  G  D  H
                                                    *  *  *
    CCAATCACAGCAAGAGCCATTGCAGCTAACGTTGGCATCATCACAGAAGGCAGCGAAACA 1620
521 P  I  T  A  R  A  I  A  A  N  V  G  I  I  T  E  G  S  E  T

    GTGGAGGACATAGCCCAGAGGAAACGCATTCCTGTTGAACAAGTCAACAAGAGGGAAGCC 1680
541 V  E  D  I  A  Q  R  K  R  I  P  V  E  Q  V  N  K  R  E  A

    CGGGCTTGTGTGATCAGCGGCGGTCAGCTCAAAGACATGACGAGCGAAGAATTAGACGAC 1740
561 R  A  C  V  I  S  G  G  Q  L  K  D  M  T  S  E  E  L  D  D

    ACGCTGCGGAACCACCCGGAGATGGTGTTTGCAAGAACGTCCCCTCAGCAGAAACTCATT 1800
581 T  L  R  N  H  P  E  M  V  F  A  R  T  S  P  Q  Q  K  L  I
                                      *        *        *
    ATTGTTGAGAGTTGTCAACGTTTGGGCTCCATCGTGGCTGTAACAGGTGACGGTGTGAAC 1860
601 I  V  E  S  C  Q  R  L  G  S  I  V  A  V  T  G  D  G  V  N
                                                    *  *  *  *
                                         ← apATP4A.elongate-R4
    GACTCTCCTGCTCTTAAGAAGGCCGACATTGGCATCGCCATGGGGATTGCCGGTTCAGAT 1920
621 D  S  P  A  L  K  K  A  D  I  G  I  A  M  G  I  A  G  S  D
    *
    GCGGCCAAGAACGCTGCAGACATGATCCTGCTGGACGACAACTTTGCTTCTATTGTCACT 1980
641 A  A  K  N  A  A  D  M  I  L  L  D  D  N  F  A  S  I  V  T

          ←—— apATP4A.elongate-R3                 Q-ATP4A-F————
    GGAGTGGAGCAGGGCCGTCTTATCTTTGACAACCTGAAGAAGTCCATTGCCTACACGCTA 2040
661 G  V  E  Q  G  R  L  I  F  D  N  L  K  K  S  I  A  Y  T  L
                                                          ▶
    ————→
    ACCAAAAACATTCCCGAGCTGACTCCATATTTGATCTACATCACCGTCAGCGTGCCTCTT 2100
681 T  K  N  I  P  E  L  T  P  Y  L  I  Y  I  T  V  S  V  P  L
      ▲  ▲        ▲
      ▶  ▼        ▼
      ^  ^        ^
         v        v
             ←———————— Q-ATP4A-R
    CCTCTGGGCTGCATTACCATCCTCTTCATCGAACTGGCCACTGACATTTTCCCCTCCGTC 2160
701 P  L  G  C  I  T  I  L  F  I  E  L  A  T  D  I  F  P  S  V
                                ▲
                                ▼        ▶
                                ^        ^
                                v
    TCTCTGGCTTATGAGAAGGCAGAGAGTGACATCATGCACCTGAAGCCCAGGAACCCACGA 2220
721 S  L  A  Y  E  K  A  E  S  D  I  M  H  L  K  P  R  N  P  R

                                                 ←———— apATP4A.
    CGTGATCGGCTGGTGAACGAACCCCTGGCTGCGTACTCATACTTCCAGATCGGCGCCATC 2280
741 R  D  R  L  V  N  E  P  L  A  A  Y  S  Y  F  Q  I  G  A  I

  elongate-R2
    CAGTC 2285
761 Q
```

Figure S3. Nucleotide (numbered to the right) and amino acid (numbered to the left) partial sequences for *C. labrosus* gastric proton pump (GenBank acc. no. MH350434). All the primers are underlined (except for qPCR oligonucleotides, which are in bold and double underlined) and arrows represent their directions. The protein belongs to the P-type ATPase subfamily IIC, subunit alpha (IPR005775) and to the Conserved Protein Domain Family cd02608 of the P-type_ATPase_Na-K_like, subfamily that includes the alpha-subunit of Na(+)/K(+)-ATPases and of gastric H(+)/K(+)-ATPase, similar to the human Na(+)/K(+)-ATPase alpha subunits 1-4. The conserved Features/Sites are the following. Feature 1, P-type ATPase signature motif, 7 residues in positions 276-282: D K [TS] G T [LIVM] [TS], highlighted in grey. Feature 2, ATP-binding site (chemical binding site), labeled with asterisks (*). Feature 3, phosphorylation site (posttranslational modification site) at position D^{276}, already highlighted in grey and labeled with an asterisk. Feature 4, phosphorylation site S (posttranslational modification site) is beyond the C-terminal end of the partial protein sequence. Feature 5, Na binding site I (ion binding site I) labeled with a solid up arrowhead (▲). Feature 6, Na binding site II (ion binding site II) labeled with a solid down arrowhead (▼). Feature 7, Na binding site III (ion binding

site III) labeled with a solid right arrowhead (►), although is missing a glutamine in a position beyond the C-terminal end of the partial protein sequence. Feature 8, K binding site I (ion binding site I) labeled with an up arrowhead (^). Feature 9, K binding site II (ion binding site) labeled with a down arrowhead (˅). Feature 10, alpha-subunit/beta-subunit interface (polypeptide binding site) is beyond the C-terminal end of the partial protein sequence, except for the 4 amino acids highlighted in white characters on black background.

```
     TCATACGCAGCTTGTGCGGGATATCATTTGCCTGAAACCGGTTCCCTTAAAGCGAAAAGC 60
     CCCCCCACCCAAAGTTCAGCCATGGAAGATGAAATTGCCGCACTTGTTGTTGACAACGGA 120
  1                       M  E  D  E  I  A  A  L  V  V  D  N  G
                                                          *  *  *
     TCCGGTATGTGCAAAGCCGGATTTGCCGGAGACGACGCCCCTCGTGCTGTCTTCCCCTCC 180
 14  S  G  M  C  K  A  G  F  A  G  D  D  A  P  R  A  V  F  P  S
     *     *     *
     ATCGTCGGTCGCCCCAGACATCAGGGTGTGATGGTGGGTATGGGCCAGAAGGACAGCTAC 240
 34  I  V  G  R  P  R  H  Q  G  V  M  V  G  M  G  Q  K  D  S  Y

     GTTGGTGATGAAGCCCAGAGCAAGAGAGGTATCTTGACTCTGAAGTACCCCATTGAGCAC 300
 54  V  G  D  E  A  Q  S  K  R  G  I  L  T  L  K  Y  P  I  E  H

     GGTATCGTGACCAACTGGGACGACATGGAGAAGATCTGGCATCACACCTTCTACAACGAG 360
 74  G  I  V  T  N  W  D  D  M  E  K  I  W  H  H  T  F  Y  N  E

     CTGAGAGTTGCCCCTGAGGAGCACCCCGTCCTGCTCACAGAGGCCCCCCTGAACCCCAAA 420
 94  L  R  V  A  P  E  E  H  P  V  L  L  T  E  A  P  L  N  P  K

         qRT-actb-Fw ------>
     GCCAACAGGGAGAAGATGACCCAGATCATGTTCGAGACCTTCAACACCCCCGCCATGTAC 480
114  A  N  R  E  K  M  T  Q  I  M  F  E  T  F  N  T  P  A  M  Y

     GTTGCCATCCAGGCTGTGCTGTCCCTGTACGCCTCTGGTCGTACCACCGGTATCGTCATG 540
134  V  A  I  Q  A  V  L  S  L  Y  A  S  G  R  T  T  G  I  V  M
              *
                                              <------ qRT-actb-Rv
     GACTCCGGTGATGGTGTGACCCACACAGTGCCCATCTACGAGGGCTACGCTCTGCCCCAC 600
154  D  S  G  D  G  V  T  H  T  V  P  I  Y  E  G  Y  A  L  P  H
     *  *  *  *
     GCCATCCTGCGTCTGGACTTGGCCGGCCGCGACCTCACAGACTACCTCATGAAGATCCTG 660
174  A  I  L  R  L  D  L  A  G  R  D  L  T  D  Y  L  M  K  I  L

     ACAGAGCGTGGCTACTCCTTCACCACCACGGCCGAGAGGGAAATCGTGCGTGACATCAAG 720
194  T  E  R  G  Y  S  F  T  T  T  A  E  R  E  I  V  R  D  I  K

     GAGAAGCTGTGCTACGTCGCCCTGGACTTCGAGCAGGAGATGGGAACCGCTGCCTCCTCC 780
214  E  K  L  C  Y  V  A  L  D  F  E  Q  E  M  G  T  A  A  S  S

     TCCTCCCTGGAGAAGAGCTACGAGCTGCCCGACGGACAGGTCATCACCATCGGCAATGAG 840
234  S  S  L  E  K  S  Y  E  L  P  D  G  Q  V  I  T  I  G  N  E

                             Q-ACTB-F ------------>
     AGGTTCCGTTGCCCTGAGGCCCTCTTCCAGCCTTCCTTCCTTGGTATGGAGTCCTGCGGA 900
254  R  F  R  C  P  E  A  L  F  Q  P  S  F  L  G  M  E  S  C  G

                                                   <----------
     ATCCACGAGACCACCTACAACAGCATCATGAAGTGCGACGTCGACATCCGTAAGGACCTG 960
274  I  H  E  T  T  Y  N  S  I  M  K  C  D  V  D  I  R  K  D  L

     -- Q-ACTB-R
     TACGCCAACACCGTGCTGTCTGGAGGTACCACCATGTACCCCGGCATCGCCGACAGGATG 1020
294  Y  A  N  T  V  L  S  G  G  T  T  M  Y  P  G  I  A  D  R  M

     CAGAAGGAGATCACAGCCCTGGCCCCATCCACCATGAAGATCAAGATCATTGCCCCACCA 1080
314  Q  K  E  I  T  A  L  A  P  S  T  M  K  I  K  I  I  A  P  P

     GAGCGTAAATACTCTGTCTGGATCGGAGGCTCCATCCTGGCCTCCCTGTCCACCTTCCAG 1140
334  E  R  K  Y  S  V  W  I  G  G  S  I  L  A  S  L  S  T  F  Q

     CAGATGTGGATCAGCAAGCAGGAGTACGATGAGTCCGGCCCCTCCATCGTCCACCGCAAA 1200
354  Q  M  W  I  S  K  Q  E  Y  D  E  S  G  P  S  I  V  H  R  K

     TGCTTCTAAACAGACTGTTCCTCCTCCCCTTCCCCGACCGAACGCCCAACTTCAGCTCTG 1260
374  C  F

     TGCAAAACAACACACGACACATCTCTCATTACACACTCAGGCGCAGAGCCTAGACGACCA 1320
     ACTCATTGGCATGGCTTCAGTTATTTTGGCGCTTGACTCAGGATTT Poly-A        1367
```

Figure 4. Nucleotide (numbered to the right) and amino acid (numbered to the left) sequences for *C. labrosus* β-actin (GenBank acc. no. MH350431) full-length cDNA. Start and Stop codons are indicated in bold and underlined. All the primers are underlined (except for qPCR oligonucleotides, which are in bold and double underlined) and arrows represent their directions. The UTRs are indicated in gray. The putative poly A signal (CUE: TTATTT) is underlined with a thicker line. The 6 motifs for PRINT PR00190 identifier for actin are box-highlight in dark gray. The 2 conserved sites IPR004001 for actin signature 1 (Prosite PS00406 ACTINS_1; [FY]-[LIV]-[GV]-[DE]-E-[ARV]-[QLAH]-x(1,2)-[RKQ](2)-[GD]) and actin signature 2 (Prosite PS00432 ACTINS_2; W-[IVC]-[STAK]-[RK]-x-[DE]-Y-[DNE]-[DE]) are boxed with a single line, whereas the actin and actin-related proteins signature (Prosite PS01132 ACTINS_ACT_LIKE; [LM]-[LIVMA]-T-E-[GAPQ]-x-[LIVMFYWHQPK]-[NS]-[PSTAQ]-x(2)-N-[KR]) are boxed with a double line. The 11 out of 12 amino acids from the conserved protein domain family cd00012 (NBD_sugar-kinase_HSP70_actin) related to the Nucleotide-Binding Domain of the sugar kinase/HSP70/actin superfamily are labeled with an asterisk (*) under the corresponding amino acids (D^{11}-N^{12}-G^{13}-S^{14}, M^{16}, K^{18}, Q^{137}, D^{154}-S^{155}-G^{156}-D^{157}).

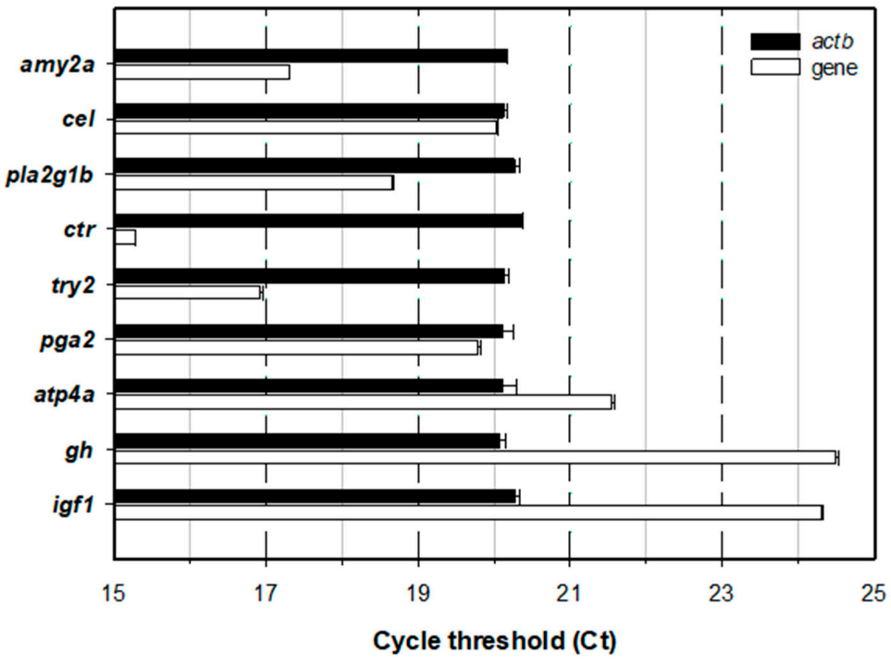

Figure 5. Comparison of cycle threshold (Ct) average values of the calibrator sample for all the studied genes, where the inter-assay (black bars lengths) and intra-assay (SEM bars) variation of the reference gene *actb* is minimal.
